# Study on the communication effect of the social livestream of cabin hospitals' construction process during the COVID-19 outbreak

**DOI:** 10.3389/fpubh.2022.978970

**Published:** 2022-11-17

**Authors:** Hualong Yu, Guang Yu, Yijun Li, Tong Li

**Affiliations:** School of Management, Harbin Institute of Technology, Harbin, China

**Keywords:** COVID-19, social media, sentiment classification, communication effect, 24-h Livestream

## Abstract

When the world is recovering from the chaos that COVID-19 creates, the epidemic is still posing challenges to the public health system and communication. However, a case of information communication during the COVID-19 outbreak can provide a reference for the current information promulgate strategy in China. In January 2020, CCTV broadcasted the construction of two cabin hospitals on a 24-h Livestream (24H-LS), creating a remarkable viewing effect. We conducted a quantitative analysis based on the number of views, social media communication, and internet search index. We collected posts and comment data of the 24H-LS audience and related topics on Weibo, using sentiment classification and word frequency analysis to study the communication effect of 24H-LS from three perspectives: perception effect, psychology, and subject issue. The results show that, first, 24H-LS has attracted extensive public attention on the Internet and social media after its launch. Second, the public's perception of the risks of the COVID-19 outbreak and its uncertainty has decreased after watching the 24H-LS. At the same time, the positive emotions of the public have been enhanced to a certain extent. Third, through subject analysis, we found that the public had high participation and strong interaction in 24H-LS, which produced collective symbols and emotions. The study shows that through 24H-LS, a new information form, the media can effectively convey important information and resolve the public's fear and anxiety.

## Introduction

The global COVID-19 pandemic has had a huge adverse impact on human society. The ongoing and epidemiologic COVID-19 both creates and complicates public health challenges (1). The COVID-19 infodemic on social media was characterized by gradual progress, videoization, and repeated fluctuations (2), which makes it more challenging to influence public opinion. Therefore, the rapid and accurate reporting of the news media, such as the development of epidemic events and the implementation of intervention policies, can enable the general public to obtain epidemic-related information promptly. It can also stabilize people's minds and emotions and promote the effective implementation of epidemic prevention measures (3). At present, governments are actively working to fight the epidemic, and many countries have achieved different goals. In order to draw on the advanced experience in anti-epidemic strategy and public communication, this paper selected the 24-h Livestream (24H-LS) related data of the construction process of the cabin hospitals during the outbreak of the COVID-19 in China as a typical case. It conducted quantitative research on the information communication effect after the live broadcast was launched. This study explored the response of 24H-LS event to public behavior, hoping to reveal the effect of 24H-LS on crisis communication in the implementation process of non-drug intervention strategies in crisis management, which can provide reference for crisis management in the era of epidemic.

As the epidemic of the COVID-19 outbreak in January 2020, Wuhan saw a sharp increase in confirmed cases, which led to a severe shortage of medical resources in designated hospitals and caused panic among the public. In response to this surge, the Wuhan government activated the Emergency Management Plan on 23 January and decided to build two cabin hospitals for COVID-19 patients: Huoshenshan and Leishenshan cabin hospitals (4). China was at its most crucial stage in fighting the COVID-19 epidemic. A series of aggressive control measures were implemented, including a total lockdown from 23 January, nationwide first-level response to major epidemics, and a full-scale operation of various non-drug interventions (5, 6). Due to late information communication and inadequate early crisis management by local governments during the early period of the epidemic (7), residents relaxed prematurely, leading to misgivings about the local government's capabilities after the outbreak (8). Fear, hopelessness, and anxiety accompanied the restrictive measures, and feelings of uncertainty, insecurity, instability and reduced self-directedness began to spread. Public information, resources for medical supplies in Wuhan, and the implementation of policies became vital.

To meet the information demand of Wuhan residents and the public for the implementation of local medical security strategies, a 24H-LS named “24h Epidemic” was launched on China Central Television Network's (CCTV) online platform for the construction of Huoshenshan and Leishenshan cabin hospitals on 27 January. Information themed by Huoshenshan and Leishenshan 24H-LS became social media's most popular anti-epidemic event (9). To reveal the impact of real and effective information communication on people's emotions and behaviors during the epidemic, we conducted a quantitative study on the communication effect of 24H-LS based on communication theory and text mining. This study aims to provide a reference for optimizing the government's new media information communication strategy for the emergency management of a major crisis.

Livestream is a new type of social media that can deliver video content in real time with many social interaction functions (10). However, 24H-LS and ordinary Livestream differ in that the latter presents the entire event for a long time without interruption and host, narrator, script, or editing, allowing viewers to comment and discuss openly. The camera is set at the event's location, and the actual scene is recorded. Moreover, it can be live-streamed through a live platform and simultaneously on various websites and social media. The earliest case of 24H-LS is the “Bergensbanen Minutt for Minutt” reported by *Time* magazine in 2009 (11). In 2013, “iPanda Videos”—a 24-h live broadcast of pandas shot by CCTV—gained many viewers both domestically and internationally. 24H-LS is characterized by a slow pace, authenticity, no intervention, online social interaction, and other attributes that enable viewers to experience a sense of presence and engagement. This reflects the openness and effectiveness in information communication during the event development process. Therefore, 24H-LS schemes integrating social media attributes create excellent viewing effects (11).

Scholars have researched the role of information communication in emergencies. And some institutions also use the existing information communication research results to assist emergency management. In 1985, Shaw proposed that governments use advanced communications technology to manage disasters in emergency management (12). Beverungen et al. analyzed the success cases of many city municipalities in Germany using the Facebook platform to establish a government-citizen communication model, laying the foundation for the effective implementation of emergency management (13). Spiro et al. studied the communication of information released by the government during the Waldo Canyon fire and constructed a model of the relationship between information content, form, and forwarding behavior (14). A study of more than 5,000 tweets in the Bordeaux flood case found that short messages can also positively affect the public's awareness of self-protection in disaster scenarios (15). Wukich analyzed the information communication of emergency management agencies in the United States, pointing out that social media can help the government strengthen crisis communication capabilities, so the government should make full use of social media to increase information communication in the stage of crisis prevention, prevention and recovery, to reduce the overall risk of emergencies (16). Min et al. analyzed 355 short videos posted on TikTok by government media during the outbreak of COVID-19 using negative binomial regression. The results showed that video length, title, dialogue loop, and content type significantly affected citizens' participation through the adjustment of emotional potency (17).

During the COVID-19 outbreak, studies have effectively promoted the application of new media technologies in the information communication strategy of governments' crisis management. A quantitative analysis of public comment data on Weibo during the early stage of the COVID-19 outbreak revealed that information on social media could be used to measure the public's attention to public health emergencies and found that the social media's response to the epidemic preceded that of the government (1). Hua and Shaw analyzed the timelines of China's key anti-epidemic actions at different stages based on data from social media platforms. They found that the critical points were the use of big data and digital technology, which promoted the effectiveness of vigorous response and governance measures suggest that the government should closely monitor social media data to improve the timing of information communication during an epidemic and adopt more direct and effective communication methods to address public anxiety (8, 18). Taking the case of the Department of Education in New South Wales' social media during the COVID-19 pandemic, Gorfinkel et al. (19) studied the role of governments' “we-media” in promoting cultural inclusion. Mat Dawi et al. (20) analyzed the image restoration strategy used by the Malaysian government on social media to promote public information communication during the COVID-19 pandemic and found that social media communication can promote public support and help build trust in times of crisis. Mansoor (21) found that the government's timely and accurate response in providing medical and health facilities during the crisis was crucial to improving the public's trust, alleviating anxiety, and obtaining public support for anti-epidemic strategies. Relevant academic studies have not been reported since 24H-LS was not applied to the government's media information communication agenda for crisis management before the outbreak of COVID-19. Since 24H-LS was not applied to government information communication before the COVID-19 outbreak, relevant academic research has not been reported. The “24h Epidemic” was a new attempt in the emergency management of epidemics.

This study quantitatively analyzed the information communication characteristics of the 24H-LS during the construction process of Huoshenshan and Leishenshan cabin hospitals based on information communication data from social media. The natural language processing technique was used to analyze the emotional evolution of the public during the 24H-LS of Huoshenshan and Leishenshan. We aim to provide a reference for the government's agenda on media strategies in crisis management during major epidemic outbreaks. This paper is structured as follows: Chapter 2 includes a case description of the 24H-LS and proposes research questions and framework. Data collection, pre-processing, and development of the research methodology are described in Chapter 3. Chapter 4 presents the empirical study and analysis results of the 24H-LS event's communication effect. Chapter 5 presents the conclusions, discussion, and future research directions.

## Case description and research questions

### A description of the event's information communication process

**Figure 1:**
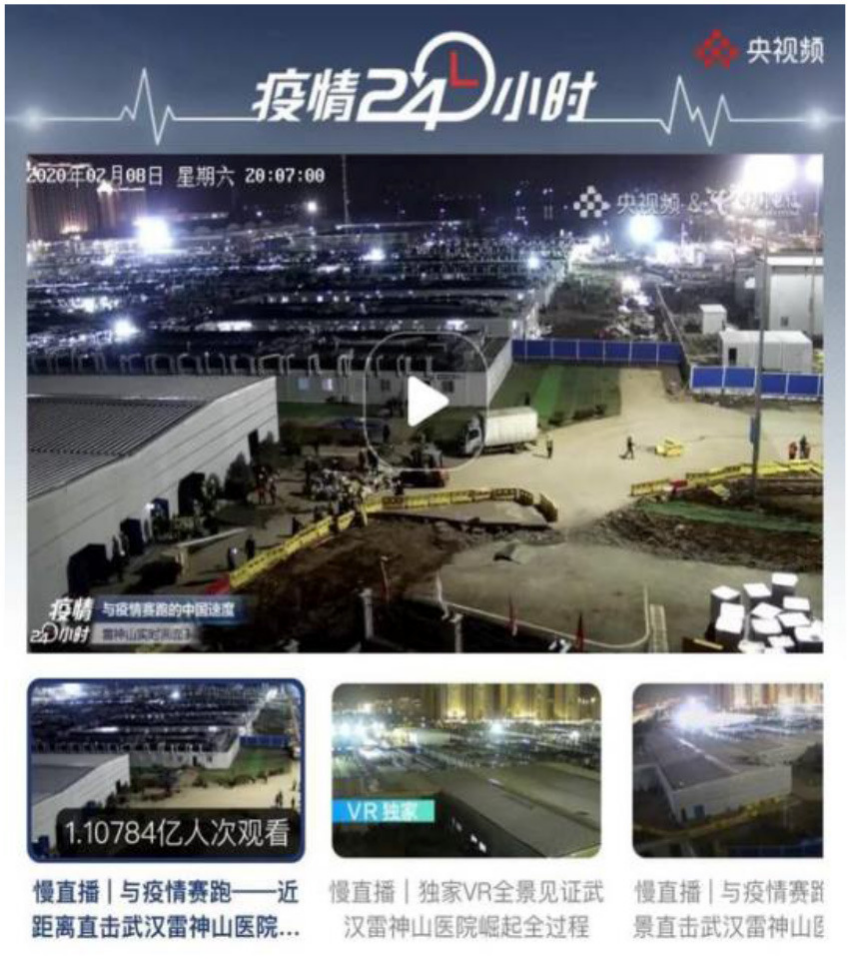
Screenshot of the 24H-LS playing interface of CCTV's online platform during the construction of Huoshenshan cabin hospital.

The construction of Huoshenshan and Leishenshan cabin hospitals was a significant measure taken by the Chinese government to fight the COVID-19 epidemic. The project was approved on January 23, 2020 and officially implemented on 2 February (22). As its central communication platform, the 24H-LS of Huoshenshan and Leishenshan used CCTV's online platform, which was implemented jointly by the ad-hoc coordination working group of CCTV and China Telecom. The platform's construction was completed on 26 January, including communication, power, and relevant equipment. On 27 January, CCTV officially launched the 24H-LS of Huoshenshan cabin hospital. With more than 30 million viewers in only 2 days, it quickly became China's most watched construction site during the COVID-19 pandemic. More than 110 million people watched the opening of Huoshenshan cabin hospital on 8 February. Figure 1 shows the 24H-LS playing interface of CCTV's online platform during the construction of Huoshenshan cabin hospital.

As shown in Figure 1, the 24H-LS event presented the actual process of Huoshenshan hospital construction, and its communication content was comprehensive, transparent, and authentic. The public could participate in the site construction with a sense of presence and immersion (23). Audiences witnessed the construction of two cabin hospitals with a common identity.

### Analysis of the 24H-LS information communication characteristics

According to the description of the 24H-LS event above, new media information communication in crisis management has its peculiarities. Based on the 5 W theory proposed by American political scientist Laswell (24), we conducted a qualitative analysis of the communicator, content, channel, audience, and effect of the 24H-LS event.

First, communicators are characterized by diversity. The initial communicator (Who) was the CCTV online platform, which released the 24H-LS simultaneously on mobile and computer terminals. Later, the Weibo accounts of People's Daily (140 million followers) and Beijing News (45 million followers) became the secondary communicators. Meanwhile, 24H-LS-related information was also forwarded on social platforms by various official media, professionals, celebrities, and other users with many followers, completing multiple rounds of secondary communication. Their social attributes have attracted the attention and participation of many netizens, producing a disruptive spread.

Second, the content of the communication (Say What) was authentic. The 24H-LS event presented the actual process of Huoshenshan hospital construction, and its communication content was comprehensive, transparent, authentic, and with no rendering. Therefore, the 24H-LS enhanced the close connection between the communicator and the audience. The public felt that they participated in the site construction, eliminating the distance and increasing the sense of presence and immersion brought about by the interaction between viewers (23). Audiences witnessed the construction of two cabin hospitals with a common identity. In this case, the audience could give full play to their subjective initiative, analyze the communication's content and creatively interpret the shots.

Third, the communication channels (In Which Channel) of the 24H-LS event exhibited multidimensional characteristics. This case was broadcasted on CCTV's online platform and through the video content of social platforms such as Weibo, TikTok, and Kuaishou. The 24H-LS event was also supplemented by traditional and online media, such as TV, radio, newspapers, web media, Weibo, WeChat, and Toutiao. The public could instantly share text, pictures, videos, and other information through various intelligent terminals, interact with people, and enact secondary transmission.

Fourth, the audience of the 24H-LS event (To Whom) was characterized by autonomous participation and group identification. According to the uses and gratifications theory, audiences use media to satisfy their needs. The primary audience of the 24H-LS during the COVID-19 outbreak included Chinese people, who were under strict epidemic precaution measures, such as cutting off social contacts and staying at home. As a result, many people felt psychological pressure, anxiety, and fear, urgently needing to obtain transparent information from the frontline of the fight against the epidemic. The 24H-LS had set up an efficient channel to instantly and directly inform the public of the progress of epidemic control. The social function of autonomous participation in communication contributed to the public's active viewing, and the 24H-LS naturally integrated people into the construction scene. The audience who joined the 24H-LS had the same interest and focus—a concern regarding the Wuhan epidemic. The same discussion topic established a shared connection, thus generating a sense of group identity.

Fifth, the effect of the 24H-LS (With What) of information communication is remarkable from the general information communication data. In communication theory, the communication effect refers to a change in psychology, attitude, and behavior caused by communication behavior with persuasion motivation (24). Real-time and honest 24 h information communication was persuasive, attracting more than 100 million people within 10 days of its launch and obtaining over 90,000,000 registered “cloud supervisors” from across the country. Moreover, the focus of the public gradually shifted to caring for construction workers and the hospitals' construction process. Participants integrated themselves into the construction scene, expecting to contribute to it and created a scene of national participation.

What are the quantified results of the information communication effect and influence of the 24H-LS, and what would be the themes, emotions, and attitudes in public communication? This study reveals an in-depth analysis of the 24H-LS communication data.

### Research questions and research framework

According to the above characteristics analysis of 24H-LS information communication based on the communication theory, the following research questions are presented from the perspective of the communication effect: (25).

**Research Question 1:** What is the communication effect of the 24H-LS measured by breadth? How much public attention did the event attract?

**Research Question 2:** From the perspective of perception effect, how did the public perceive risk during the 24H-LS event?

**Research Question 3:** From the psychological perspective, how did the public emotional state change during the 24H-LS event?

**Research Question 4:** During the 24H-LS event, what subject issue does the public express under different emotions?

To explore the information communication effect of the 24H-LS event, we comprehensively collected the information communication data of 24H-LS-related topics and the comment data of participants on social media. The research flow is described as follows:

- We established the measurement method of the event's information communication on social media to analyze the communication influence, that is, the audience breadth, including the number of views, network search index, and social media influence.
- We used the natural language processing technology to analyze the emotional changes and subject evolution of 24H-LS-related topic participants in the online interaction of the 24H-LS with the construction of Huoshenshan cabin hospital, as well as the psychological and attitudinal effects on 24H-LS audiences.
- We analyzed the participants' behavior patterns through the text mining results of Weibo and its comment data, and we quantitatively analyzed the effect of information communication of the event in epidemic crisis management according to the empirical research results.

**Figure 2:**
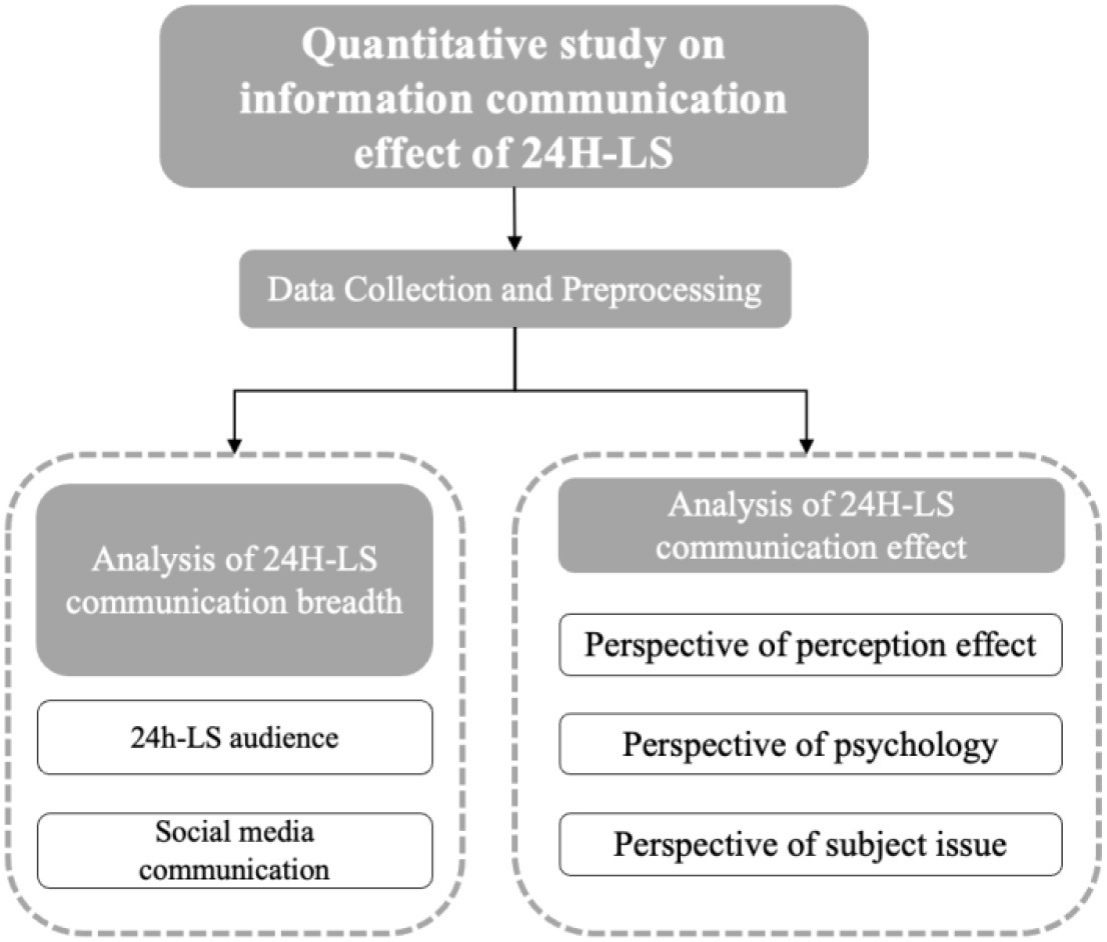
The research framework.

Figure 2 presents the research framework.

## Data and methodology

### Data collection and pre-processing

#### The data of 24H-LS communication breadth

We obtained screenshots from CCTV's online platform throughout the 24H-LS of Huoshenshan hospital's construction, from which we recorded the number of views during the 10-day 24H-LS. Meanwhile, we selected Weibo, the largest social media platform in China, as the data collection platform in this study to analyze the spread of the event on social media. First, we determined the keywords of the relevant topics, such as “Huoshenshan,” “Slow live streaming,” “24 h live streaming,” “cloud monitor,” and so on. Second, we determined the data acquisition fields according to the experimental needs, number of comments, likes, and retweets. Third, we used a web crawler to collect Weibo data from January 24 to February 11, 2020. Finally, we cleaned data to eliminate noisy data unrelated to the event.

#### User comment data of 24H-LS-related topics

We collected user comments on Weibo data in Section The data of 24H-LS communication breadth to study the participants' cognitive effects and psychological state in the 24H-LS. Owing to a large amount of Weibo data in Section The data of 24H-LS communication breadth, we selected Weibo posts with the top 10% of comments as the sample of comments collection, of which the comment number accounted for 80% of the total. We collected user comment text data from the sample using a crawler.

### Analysis method of social media information communication characteristics

To perform a quantitative analysis of 24H-LS communication, we analyzed the communication characteristics from the breadth of information communication according to the 5 W theory (26).

Breadth measures the extent to which an event attracts the audience, which is crucial for the event to spread and influence the public. This breadth is measured by the size of the audience and the number of retweets. The research object of this study was 24H-LS, of which the breadth was measured by the number of daily views and related internet and social media posts, including the following indicators:

(1) Number of 24H-LS views *V*_*n*_: This indicator describes the number of viewers watching 24H-LS on CCTV's online platform, which was the cumulative viewing number on day n. This study obtained the growth trend of *V*_*n*_ through the statistics of webpage screenshots in different moments.
(2) Social media communication index *Y*_*n*_: This indicator describes the number of Weibo posts related to constructing the two cabin hospitals on day *n*. *Y*_*n*_ measured the spread trend of the cabin hospital's construction event.

### Measurement method of risk expression of audiences

Information is an essential factor affecting the perception of risk. When 24H-LS-related information was spread on social media, a large amount of interactive information was generated, which is the data basis of the participants' risk perception measurement study. In the early days of the outbreak, panic and fear gripped the population. We used text mining to measure the public's perception of risk. Therefore, we observed 24H-LS viewers' comments about “uncertainty” and “dread” to determine their perception of risk. The method includes three steps: First, we construct the risk perception dictionary of the two dimensions of uncertainty and dread; second, we match Weibo's comment text with the risk perception dictionary. Finally, we counted the frequency of risk perception words occurring daily, which is the risk perception value.

We first consider the dread aspect of risk perception (27), including whether the epidemic is under control, widespread, and deadly. Second, we considered the unknown aspect, including whether the outbreak was familiar to the public, whether it was known to science, and whether the virus could be monitored. Based on reference to the CLIWC dictionary (28) and texts in the data set of this study, a dictionary of risk perception was manually established, covering 712 terms of dread and 74 terms of unknown. Examples are shown in Table 1.

**Table 1:** Example of risk perception dictionary.

**Dictionary of dread**			**Dictionary of uncertainty**		
Scary	Scary to death	Deadly panic	Doubt	Not understand	Perhaps
Shudder	Uneasy	Afraid	What to do	Hide	May
Horrified	The willies	Terrible	Suspected	Not sure	Probably
Creepy	The controls	Fear	What if	Not clear	Volatility
Crisis	No escape from fate	Dangerous	Is there any	Uncertain	Shock
Palpitations	Trembling	Critical	Unknown reason	Unsharp	Turmoil
Fearsome	Around	Terrorist	Don't have a clue	Not exclude	Unstable
Unsettled	Awe	Panic	Don't know	Not rule out	Change
Beat	Scared	Dread	Unpredictable	Ambiguity	Variable
Shockingly	Half dead with fright	Worry	Will or will not	Unknown	Alteration

We matched the Weibo posts with the terms of dread and uncertainty, then counted the number of Weibo posts containing two terms on day *n* as the public risk perception value of the day, which is *RP*_*n*_.

We used *RP*_*n*_ to measure the risk word expression on social media of the day. The risk expression tendency of the public on day *n* is the ratio of the number of microblogs with dread and unknown tendency on day n to the total number of microblogs with sample *W*_*n*_ on day *n*, which is represented by β_*n*_.

### Methods of audience emotion analysis in information communication

Emotion is an essential psychological behavior of human beings. To study the public psychological status of the 24H-LS event, we conducted an emotional analysis of the user comments data in Section User comment data of 24H-LS-related topics. BERT is a new sentence-level language pre-training model (29), and training are based on left-right and contextual information, which is suitable for Weibo comments' text data. Therefore, we selected Chinese-RBT3 pre-trained model to achieve the emotion calculation task of large-scale Chinese text.

Through the observation of the data, the public's emotional tendencies are generally manifested in three categories: positive, neutral, and negative. We define comments that show emotions, such as reassurance, joy, trust, and praise, as positive, comments that show sadness, fear, and anger as negative, and comments that do not express apparent emotions as neutral.

First, two annotators simultaneously tri-categorize randomly selected comments. When the two annotation results of the comment are the same, the result is passed. Second, we labeled the randomly selected comments in three categories and balanced the number of the three labels. Third, we divided the labeled samples into training and test sets at a ratio of 9:1. We obtained 9,800 labeled data of 24H-LS event comments, among which 3,200 were training sets and test sets.

Based on the Chinese-RBT3 pre-training model, we obtained the 24H-LS emotion classification model through training. The accuracy rate of the model is 84.06% on the test set, and the precision, recall, and f1 value rate are all above 78%, as shown in Table 2. We used the model to classify all Chinese comment data into three categories of emotion (positive, neutral, and negative) to observe people's attitudes and emotional tendencies in the comments.

**Table 2:** Chinese-RBT3 pre-training model test results.

	**Precision**	**eval_recall**	**f1_score**
Positive	0.8211	0.8279	0.8245
Neutral	0.8614	0.8512	0.8563
Negative	0.8065	0.78125	0.7937

### Method of audience subject extraction

To understand the source of people's emotions, analyzing the subjects of the comments is necessary. Based on the data in Section User comment data of 24H-LS-related topics, we conducted word frequency statistical analysis and keyword extraction.

The word frequency analysis process is as follows: First, we selected the HIT stop word list, commonly used in Chinese segmentation, to remove meaningless words. And we selected 2020 epidemic-related vocabulary to establish a custom segmentation dictionary. Second, all comment text was processed using python jieba Chinese word segmentation algorithm, with the stop word list and the custom segmentation dictionary. Third, we repeated the above steps until the segmentation output was completed. Finally, we used the frequency measurement statistics through the word segmentation results to obtain the subjects of public comments in the 24H-LS event.

## Results

### Analysis of 24H-LS communication breadth

According to the research method in Section Analysis method of social media information communication characteristics, we analyzed the communication breadth of the 24H-LS audience and related information on social media.

#### 24H-LS audience

**Figure 3:**
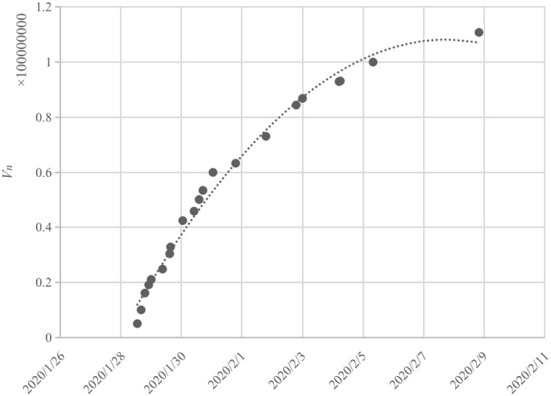
Daily distribution of *V*_*n*_.

The number of views, *V*_*n*_, was used to measure audience numbers. Because there was no direct way to obtain the real-time viewing data of CCTV's online platform, we counted the release time of daily news. We recorded the cumulative viewing number displayed in the screenshots. The daily trend of *V*_*n*_ is as shown in Figure 3.

In Figure 3, 24H-LS attracted significant attention since January 27, 2020, and the cumulative number of viewers grew at a swift pace during the first 3 days, with an average daily increase of ~20 million. Although the growth rate slowed after 31 January, the total number of viewers reached 100 million on 5 February and 110 million on 8 February, when the livestream ended. In addition to CCTV's online platform, the 24H-LS of Huoshenshan hospital construction also received significant attention on other platforms. For example, on Weibo, People's Daily's “round-the-clock live broadcast # Direct Coverage of Huoshenshan Hospital Construction Site” was viewed by 31.72 million people, and “direct construction process of the Beijing news | make Huoshenshan hospital supervisor # # together” was viewed by 310 thousand people.

#### Social media communication

We found that the influence of the 24H-LS event on the internet was not limited to 24H-LS audience. The media created topics related to the 24H-LS event, which then caused extensive public discussions on social media. The topic coverage time range was January 24 to February 13, 2020. According to the research method in Section Analysis method of social media information communication characteristics, the total amount of information ∑ *Y*_*n*_ on the Weibo platform was calculated as 3,568,751.

**Figure 4:**
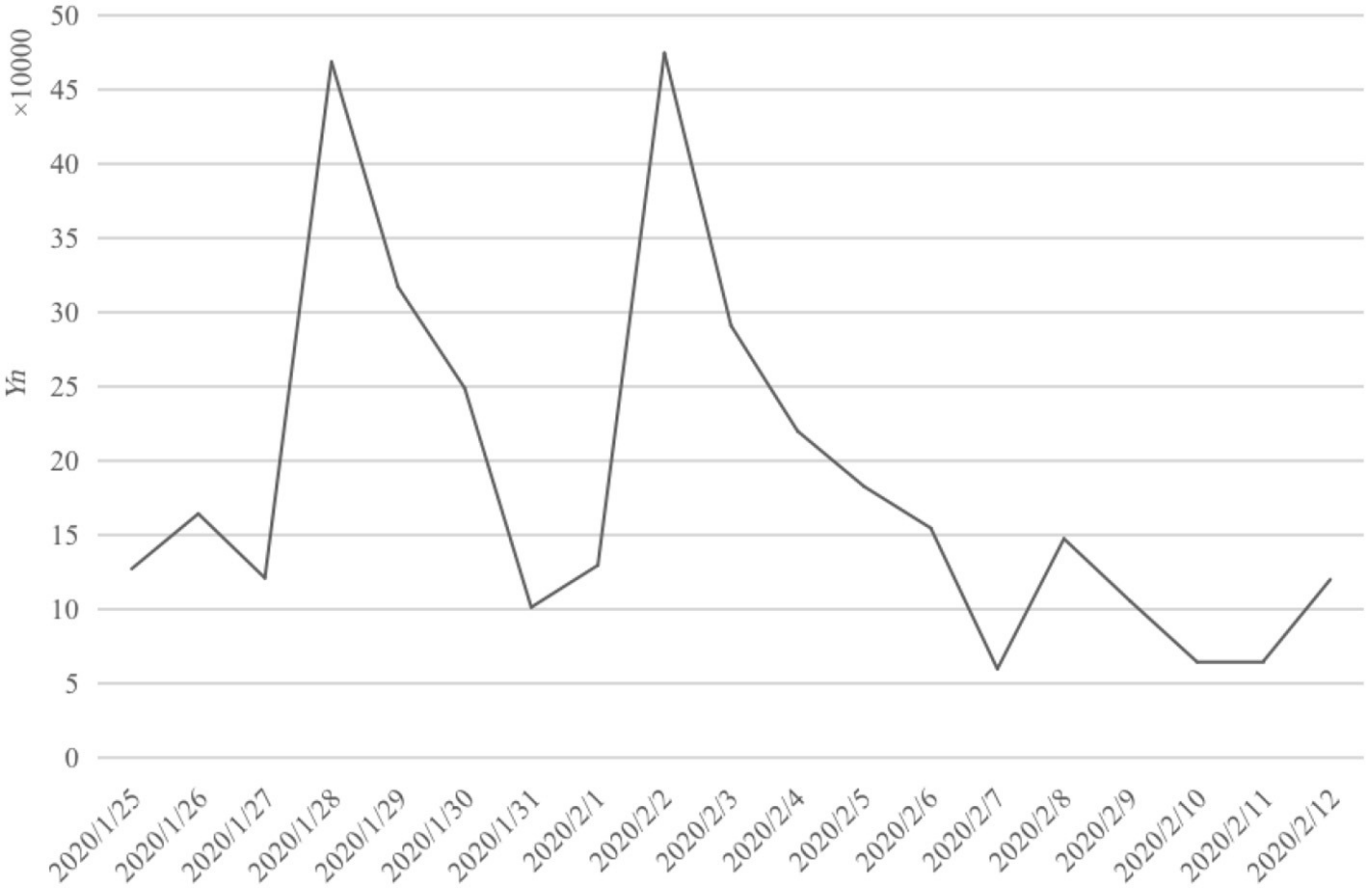
Communication breadth results.

The trend of *Y*_*n*_ is shown in Figure 4. We found that the event had attracted much attention since its release, and the *Y*_*n*_ remained at around 100,000 from 25 January to 27 January. After the broadcast started on 28 January, *Y*_*n*_ reached a peak of 450,000 for the first time and fell. On 2 February, Huoshenshan Hospital was officially constructed and delivered, *Y*_*n*_ reached a peak of 450,000 again. *Y*_*n*_ began to decline after the end of the 24H-LS on 3 February and fell below 150,000 on 6 February. Since 12 February, a series of patients were discharged from the hospital, and the number of the posts increased slightly again.

### Risk perception analysis

**Figure 5:**
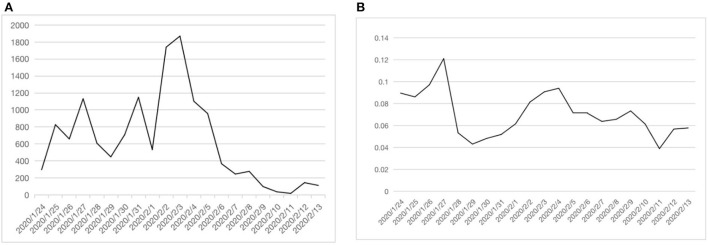
Trends of people's risk perception. **(A)** Risk perception value *RP*_*n*_. **(B)** Risk perception tendency β_*n*_.

According to the risk perception calculation method in Section Measurement method of risk expression of audiences, we analyzed the user comment data of 24H-LS-related topics in Section User comment data of 24H-LS-related topics. We calculated the risk perception from comments on Weibo, got *RP*_*n*_, and drew the time trend chart of the amount of risk perception. Then β_*n*_ was calculated and the trend chart of risk perception tendency was drawn to measure the change of risk perception degree of the public, as shown in Figure 5 below.

As can be seen from Figure 5, from January 24 to January 27, the hospital was under construction, 24H-LS had not yet started, β_*n*_ was at a high level of around 0.1. On January 28, after 24H-LS began, there was a significant drop in β_*n*_. On February 2, the hospital was built and began to accept COVID-19 patients, which brought new uncertain factors and risk points. Both *RP*_*n*_ and β_*n*_ increased significantly. After the hospital worked normally, *RP*_*n*_ and β_*n*_ decreased to a lower level. In general, as 24H-LS improved the information transparency and openness of the hospital construction process, the trend of public risk perception fluctuated.

### Emotion analysis

We performed an emotion analysis with the comment data of 24H-LS-related public topics on Weibo. After calculating the emotion tendency values of 185,308 comments, the results of the three categories of emotions were obtained (Table 3). “Neutral” accounted for the majority of the comments, and the positive emotions in the comments were significantly higher than the negative ones (fear, sadness, and anger).

**Table 3:** Emotion distribution statistics of comment data of 24H-LS-related topics on Weibo.

	**Quantity**	**Ratio**
Positive	71,075	38.5%
Neutral	96,154	51.8%
Negative	18,079	9.7%
Total	185,308	100%

**Figure 6:**
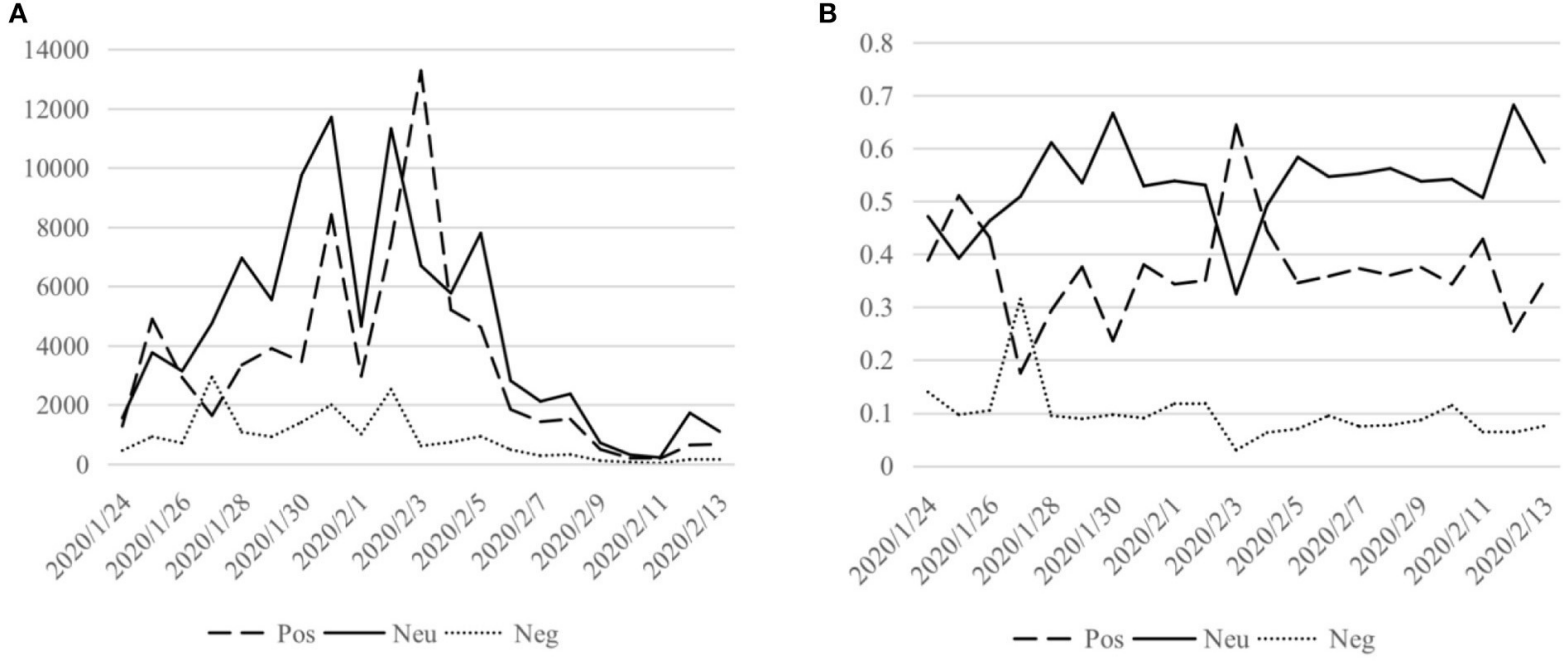
Trend of comments in three emotions on 24H-LS-related topics. **(A)** Quantity of comments. **(B)** Ratio of comments.

We counted the number of comments classified by emotion according to the comment release date, and the statistical results from January 24 to February 13, 2020 were taken to show charts of the quantity and proportion of comments in three emotions (positive, neutral, negative), respectively, as shown in Figure 6.

On January 26–27, before the 24H-LS was broadcast, the number of negative comments increased from 712 to 2,946, accounting for 10.5–31.5%, and the number of positive comments dropped from 2,929 to 1,639, accounting for 43.2–17.5%, reflecting the instability of public sentiment at that time. After the official launch of the 24H-LS on January 28, the number of negative comments was always between 900 and 2,000 from January 28 to 31, and the proportion remained below 10%; At the same time, the number of positive and neutral comments showed a significant increase, reaching 8,425 and 11,720 on January 31. On February 3, the construction of Huoshenshan Hospital was completed, and the number of positive comments reached a peak of 13,286, accounting for 64.5% of the total number of comments on the day, far higher than the average of 38.5%, and negative comments accounted for only 3.0%, indicating that the overall public sentiment on this day was positive and showed strong confidence. Since February 6, Huoshenshan Hospital has entered a period of stable operation. The number of negative comments has dropped to <500, the proportion has consistently remained below 12%, and the number of positive and neutral reviews has gradually fallen back to stabilize.

### Analysis of topics of public concern

In order to study the influence of 24H-LS event on the topic of public concern (Question 4), we use the research methods in 3.5 to conduct topic analysis on user comments perceived as risks and user comments under different emotions.

**Figure 7:**
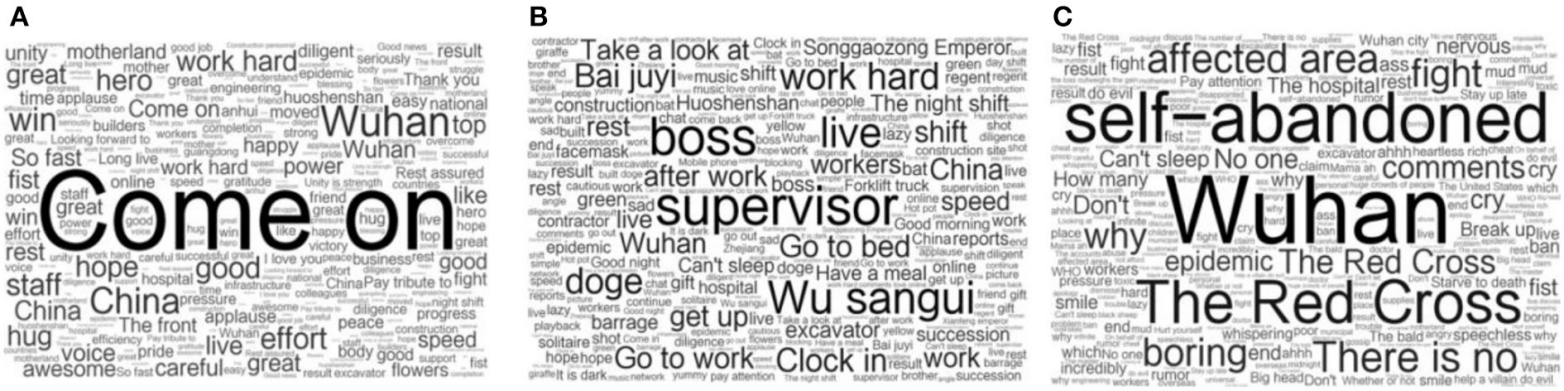
Cloud map of keywords in comments with different emotional tendencies. **(A)** Comments with positive emotions. **(B)** Comments with neutral emotions. **(C)** Comments with negative emotions.

Based on the interactive comment data of the 24H-LS audience, we conducted a topic analysis for positive, neutral, and negative comments. We obtained words with high frequency in the discussion, removed meaningless words, and counted the frequency of keywords. Finally, we created a word cloud map based on the statistical results (Figure 7).

Figure 7A presents the theme of positive comments. The themes of the comments included praising the construction speed of Huoshenshan Hospital, encouraging hospital builders, and expressing confidence in fighting the epidemic. High-frequency words include “come on,” “good,” “work hard,” and so on.

Figure 7B presents the theme of neutral comments; it shows that the audience deeply participated in the construction of Huoshenshan Hospital in the 24H-LS. On the one hand, most 24H-LS audience were at home and voluntarily participated in cloud monitoring activities by watching live broadcasts; keywords such as “take over,” “go to work,” “go off work,” and “report” appeared in the comments, and the audience formed a strong interactive relationship and mutual companionship. On the other hand, audiences used homophonic, personification, and other ways to create many “pet names” for the equipment under construction, such as forklift as “little fork”, concrete mixing truck as “Songhuizong” and high-rise crane as “Songgaozong”. CCTV.com also launched a reward function for these devices, making the boring 24H-LS interesting.

Figure 7C shows the theme of the negative comments. It appears that the public did not stand on the opposite side of the builders of Huoshenshan and rarely made negative comments on the construction of Huoshenshan Hospital itself. They mainly focused on three aspects: the lack of confidence in fighting the epidemic, which is manifested in the words “pressure” and “disappointment”; other aspects of epidemic emergency management that attracted significant attention, such as “doing whatever they want,” “black sheep,” and “being rich and heartless”; and social emotions in online communication, that is, disputes between people due to different opinions through words such as “three questions do not know,” “blind,” and others.

The analysis of the above research problems revealed that the 24H-LS surmounted the physical space limitations and made the construction process of the two cabin hospitals visible to countless audiences who could not visit the site in a limited time and space. Through the 24H-LS, audiences could intuitively feel the process and results of the builders' hard work, which also enabled the implementation of critical strategies for the emergency management of major epidemics to be more open and transparent. In the critical period of fighting the epidemic, 24H-LS allowed the public to access important information in time, achieved good communication effects and played a role in stabilizing public sentiment, and improving the government's credibility.

## Conclusion and discussion

### Conclusion

(1) From the perspective of the breadth of information communication, we draw the conclusion by integrating the number of views of the 24H-LS and the quantity of Weibo-related information. In terms of the total number of views, the cumulative number of views of the 24H-LS exceeded 1 million, and the total amount of relevant information on Weibo exceeded 3,560 thousand. The 24H-LS became one of the most important and influential information communication events on the Chinese internet during the epidemic. And the event received significant, continuous exposure and public discussion. In terms of variation trends, the amount of social media communication of 24H-LS on topics related to hospital construction is also eye-catching. On the one hand, the number of Weibo amount increased to varying degrees on the day and the next day since the 24H-LS started, reaching its peak on 3 February when the hospital was completed. On the other hand, due to the uninterrupted real-time information communication properties of the 24H-LS, the hospital construction process achieved sustained exposure and public participation, which is a departure from the widespread phenomenon of the rapid growth and fading of popularity in most Internet events.
(2) From the perspective of the perception effect, the study found that although the public's perception of risk and uncertainty still exists during the 24H-LS, the concern declined significantly after the 24H-LS started, according to the temporal analysis of the risk perception value. As mentioned earlier, information about the severe strain of medical resources brought by the outbreak of COVID-19 had increased public's panic and anxiety, and the delayed response and insufficient information transparency of the government had eroded public's trust (8). According to Cognitive Consistency Theory, traditional unilateral information with obvious direction and attitude gradually become ineffective (30), which results in the block of essential epidemic information communication chains. However, with the properties of no cutting and processing and presenting the event's development, the 24H-LS provided audiences with a real “sense of presence”. It helped the public to have transparent access to the real situation of the construction of the cabin hospitals, which let people see the hope of solving the problem of medical resources shortage and reduced the public's inner panic and anxiety to some extent.
(3) From the psychological perspective, in the analysis results of the public comment emotion and the subject of the direct participation audience of the 24H-LS and its related Weibo information, an essential feature of the 24H-LS was the robust interactivity of the audience. According to the interactive ritual theory, participants in interactive rituals have four typical experiences: group unity, emotional energy, group symbol, and moral sense (31). During the construction of two cabin hospitals, the participants in the 24H-LS obtained a common identity as “cloud supervisors” of Huoshenshan hospital's construction, which enabled them to find an identity and form a virtual solidarity group with the same action goals and expectations. In this group, the participants have the same action goals and expectations: the hospital is successfully completed as scheduled, the patient is healed, and the epidemic is alleviated. As a result, a shared positive emotional energy was generated. Comments on related topics are generally biased toward positive emotional expressions, with positive and neutral emotional comments accounting for 90.3%. During the 24H-LS, although the overall number of comments increased significantly, the emotional changes in the commenters were not significant.
(4) From the perspective of the public subject issue, we found that the public was primarily concerned about hospital patients' rescue after admission, as well as the concern and uneasiness about the safety of medical staff and builders. Secondly, in the thematic analysis of different emotions, it is found that the public was immersed in the construction participation and was interested in including numerous group symbols and nicknames for industrial equipment. This behavior demonstrated the participants' ongoing communication of emotions, beliefs, and values. Through “cloud supervision” on the 24H-LS platform, audiences not only obtained the satisfaction of their information demands but also gained spiritual comfort and moral resonance; they encouraged, supervised, and respected each other to maintain common justice and to identify with group symbols. Combined with the results of the topic mining of negative information discussed by participants in the 24H-LS, we also found that in the comments, the public mainly generates emotional vents about some negative events during the COVID-19 outbreak, but not negative emotions about the construction process.

### Implications

Based on the findings of this paper, we can get insights into major crisis management.

Firstly, during the COVID-19 outbreak in China, the rapid construction of cabin hospitals such as Huoshenshan cabin hospital as a vital emergency management measure of the government played a role in responding to the increasing number of infection cases caused by the rapid spread of the virus and significantly reduced the harm to people's lives caused by the catastrophic events of sudden infectious diseases.

Secondly, the government's mainstream media CCTV adopted the 24-h uninterrupted Livestream to push real-time and transparent hospital construction information to various media channels, which not only realized the high integration of traditional media and new media but also attracted hundreds of millions of audiences to watch. The 24H-LS brought much relevant information communication through the Internet and social media and built a ritualized communication space with virtual reality for the Chinese who implemented home isolation during the epidemic. This greatly met the public's emotional needs and value recognition during the COVID-19 outbreak.

Thirdly, as a new form of media communication, the 24H-LS allowed the hospital construction process to obtain a sustained high degree of public attention, which had an impact on the subject of public concern while calming people. Our study shows that the 24H-LS construction of Huoshenshan and Leishenshan cabin hospitals reduced the public's perception of the risks and uncertainties of the epidemic. The solid interactive nature of 24H-LS made it easy for the audience to get empathy, support, and encourage each other emotionally, and form a positive atmosphere of public opinion in times of crisis.

Most countries worldwide are recovering from the COVID-19 pandemic, but China is still in a state of strict epidemic containment due to population and management policies. China's epidemic control policy is still rigorous in 2022, and the “Dynamic Zeroing Policy” has a lasting impact on the production and life of the Chinese, but the government has not maintained openness in the decision-making and management process. For example, the lockdown in Shanghai in April and May and the lockdown in Chengdu in August and September have both seen untimely policy releases and opaque information disclosure. Unfortunately, similar events, such as the construction process of the two hospitals in 2020, have not reappeared in China's epidemic control process. Therefore, even in 2022, this study will still have special enlightenment on China's emergency management of the corona epidemic.

### Limitations and prospects

In this study, preliminary exploratory research was conducted on the information communication effect of the 24H-LS during the construction of Huoshenshan and Leishenshan cabin hospitals. Although some results were obtained, the study has inevitable limitations.

First, viewers' interactive data (including comments on live subtitles and onsite discussion of participants) on the Livestream platform are protected and cannot be obtained retrospectively. Therefore, we obtained data on related topics from Weibo, the largest streaming platform outside the official live streaming. The inability to mine the behavior of live viewers in the live streaming room is the most regrettable factor of this study.

Second, we found in our research that the negative comments from the public touched on some important public issues during the epidemic, such as the lockdown, the Red Cross incident, and the Wuhan Virus Institute incident. This raises important questions for our follow-up research: the relationship between government policy and media agenda-setting based on the theory of online agenda-setting and ritual interactive chain and its role in the public agenda, which is undoubtedly an excellent research topic.

Finally, although this paper observes the sentiment of user comments on related topics during 24H-LS, the impact of 24H-LS o public sentiment has not been strictly verified by causality, which is a crucial problem that needs to be solved in the next stage of research.

These research results will provide a valuable reference for the government to use new media technology to communicate relevant information on emergency management of major crisis events and improve the government's credibility.

## Data availability statement

The raw data supporting the conclusions of this article will be made available by the authors, without undue reservation.

## Ethics statement

Ethical review and approval was not required for the study on human participants in accordance with the local legislation and institutional requirements. Written informed consent from the participants was not required to participate in this study in accordance with the national legislation and the institutional requirements.

## Author contributions

HY, GY, and YL contributed to conception and design of the study. HY and TL performed the statistical analysis. HY wrote the first draft of the manuscript. GY and HY contributed to manuscript revision, read, and approved the submitted version. All authors contributed to the article and approved the submitted version.

## Conflict of interest

The authors declare that the research was conducted in the absence of any commercial or financial relationships that could be construed as a potential conflict of interest.

## Publisher's note

All claims expressed in this article are solely those of the authors and do not necessarily represent those of their affiliated organizations, or those of the publisher, the editors and the reviewers. Any product that may be evaluated in this article, or claim that may be made by its manufacturer, is not guaranteed or endorsed by the publisher.
